# The need for more holistic immune profiling in next-generation SARS-CoV-2 vaccine trials

**DOI:** 10.3389/fimmu.2022.923106

**Published:** 2022-09-20

**Authors:** Robert L. Murphy, Eustache Paramithiotis, Scott Sugden, Todd Chermak, Bruce Lambert, Damien Montamat-Sicotte, John Mattison, Steve Steinhubl

**Affiliations:** ^1^ Northwestern University, Evanston, IL, United States; ^2^ Feinberg School of Medicine, Northwestern University, Chicago, IL, United States; ^3^ CellCarta, Montreal, QC, Canada; ^4^ Arsenal Capital, New York City, NY, United States; ^5^ physIQ, Chicago, IL, United States

**Keywords:** immune profiling, SARS – CoV – 2, vaccine trial design, vaccines, COVID - 19, cellular immunity

## Abstract

First-generation anit-SARS-CoV-2 vaccines were highly successful. They rapidly met an unforeseen emergency need, saved millions of lives, and simultaneously eased the burden on healthcare systems worldwide. The first-generation vaccines, however, focused too narrowly on antibody-based immunity as the sole marker of vaccine trial success, resulting in large knowledge gaps about waning vaccine protection, lack of vaccine robustness to viral mutation, and lack of efficacy in immunocompromised populations. Detailed reviews of first-generation vaccines, including their mode of action and geographical distribution, have been published elsewhere. Second-generation clinical trials must address these gaps by evaluating a broader range of immune markers, including those representing cell-mediated immunity, to ensure the most protective and long-lasting vaccines are brought to market.

Circulating anti-SARS-CoV-2 antibody levels naturally drop over time (1). This does not mean that antibody-mediated immune protection has disappeared, as memory B cells remain in circulation ready to reactivate upon new SARS-CoV-2 exposures (1–3). It does mean that antibody titers cannot be used as a marker of continuous long-term protection. Moreover, measuring antibody titers or antibody-mediated neutralization provides little indication of vaccine efficacy against novel variants of concern (VoC). The spike protein used by SARS-CoV-2 to enter cells, which is the major target of antibody-based immunity, is subject to mutations that allow the virus to escape detection by antibodies generated by vaccines (4). It is currently extremely difficult to determine the extent to which antibodies generated against previous infections or vaccinations will recognize spike proteins for new VoC. These gaps in understanding brought about by measurements of antibodies alone have caused real-world consequences. Immunological protection against infection appears to wane more rapidly than anticipated (5), and previously non-existent VoC have been shown to circumvent vaccine-induced immunity against the ancestral strain, although morbidity and mortality are still reduced (6, 7). Finally, some people living with immune-compromising conditions will not make antibodies correctly or in effective quantity [see discussion in (8)]. Antibody-deficient people may still be afforded vaccine-induced protection *via* the cell-mediated branch of the immune system (9), but focusing exclusively on antibodies as the measure on vaccine success leaves this possibility unevaluated, and consequently, many immunocompromised people have little or no indication of their true vaccine protection status.

These three inadequacies—waning protection, lack of robustness to mutation, and lack of efficacy in immunocompromised subgroups— have resulted in many policy recommendations to perform 3^rd^, and even 4^th^ booster campaigns at 4–6-month intervals. Vaccine boosters at such regularity are a poor solution for many reasons. Logistics and operational costs of repeated global vaccination campaigns are not sustainable. First-generation vaccination programs required heavy subsidies from government bodies to bring them to market and render them affordable to all (10). Updating current vaccine formulas to address novel emergent VoC is not a viable long-term strategy either, because the period of dominance for any particular VoC may be too short, not allowing sufficient time to revise and distribute new vaccine formulas before the bulk of mortality/morbidity caused by the VoC has passed. Also, as discussed above, with the current antibody-centric metrics of vaccine effectiveness, some immunocompromised people may never be regarded as reaching sufficient protection regardless of the number of administered vaccine boosters, although this might not necessarily be the case. Given the unsuitability of frequent vaccination as a long-term solution, second-generation vaccine trials should strive to address these three inadequacies by broadening their metrics of vaccine success to include measures of cell-mediated immunity along with measures of antibody titres.

Second-generation vaccines should be designed at the outset with the express intent of maximizing their duration of effectiveness against all SARS-CoV-2 variants as well as increasing their resistance to novel viral mutations. This may be accomplished in part by designing vaccines to maximize the contribution of cell-mediated immunity. Here, cues may be taken from the field of immune-oncology where many vaccination strategies attempt to favor cell-mediated immune responses. Strategies include rational design of MHC-restricted epitopes as a part of vaccine development; targeting peptide vaccines to intracellular compartments within antigen presenting cells *via* viral, bacterial, liposomal, chemical, or peptide-based vectors; and specific adjuvate formulations (11). Route of vaccine entry is also an important consideration, as direct stimulation of mucosal cell-mediated immunity may result in stronger cell-mediated immune responses at the main sites of viral entry (12).

Recent evidence suggests that cell-mediated immune responses may help address all three major challenges in parallel: 1) they are longer lasting than antibody-mediated responses (13), 2) they have the potential to target a larger repertoire of viral antigens—including internal antigens not expressed at the virion’s surface—making them less suspectable to escape mutations (14–18), and 3) they can provide protection in people with deficiencies in antibody-mediated responses (19–21). Given these advantages, cell-mediated vaccines are currently being developed in small animal models (22, 23). T cell vaccine epitopes injected into antibody-deficient transgenic mice have confirmed the generation of protective vaccine-specific CD8+ T cells with enhanced polyfunctional cytokine production capable of developing into effector-memory phenotypes (22).

Second-generation vaccine developers also face important challenges in designing clinical trials. The worldwide topology of the anti-SARS-CoV-2 immunological landscape has greatly changed since the time of the first-generation clinical trials. Most participants in first-generation trials had no prior SARS-CoV-2 immunity. In contrast, second-generation vaccine trials will enroll subjects with varying degrees of previously existing anti-SARS-CoV-2 immunity. At the time of this writing, approximately 66% of the world has already received partial or full vaccinations in some form or another (24), and 481 million cases of COVID-19 have been confirmed worldwide (25), although infection rates are likely under-estimated due to under-diagnosis and lack of reporting of home test results. Pre-existing immunity to natural SARS-CoV-2 infection may vary depending on individual and population-level immune statuses (20, 21) and/or the type of vaccine received (26), which can be further complicated by “mix-and-match” vaccination (27, 28). If participants are simply enrolled *via* population demographics, differing levels of immune competence and pre-existing anti-SARS-CoV-2 immunity will confound the clinical trial data. To control for these variables, the immunological status of trial participants must be evaluated at trial enrollment. This will allow segmentation of enrollees into distinct cohorts at study outset, and will ensure sufficient statistical power for specific subpopulations of interest. Self-reporting of previous infection is been an unreliable means of cohort segregation because many people experience asymptotic COVID infection and may have imperfect recollection of their vaccine and infection history. Immune profiling at trial screening should therefore aim to be holistic, focusing on both antibody and cell-mediated immune responses.

Although humoral immunity is relatively straightforward to assess, cell-mediated immunity involves the complex interplay of many effector cell types (29). It is also worth noting that the signature of pre-existing cell-mediated immune protection for healthy people will not necessarily be the same as for those living with immunocompromising conditions. As such, careful consideration must be taken when selecting the biomarkers of anti-COVID cell-mediated immunity both pre and post vaccination. At a minimum, CD4+ T cell function should be measured. CD4+ T cell function is the foundation of most cell-mediated and humoral effector activities, and therefore it is the most likely to remain consistent between fully immune competent and immunocompromised persons. Indeed, CD4+ T cell help is associated with protection conferred through antibodies (30) or effector T cells (31), and has been correlated with protection in people with suppressed immune systems (32). More specific correlates of vaccine-induced cell-mediated immunity are yet to be defined. Some clues can be drawn from natural infection and animal studies. These include reduced SARS-CoV-2 specific Tregs (33, 34), CD4+/CD8+ cell ratios (35), the presence of SARS-CoV-2 specific memory T cells in the lungs (36), and serum sST2 levels (37).

Ultimately, the correlates of vaccine-induced cell-mediated immune protection must be characterized within clinical trials for vaccines designed to generate cell-mediated immune responses. This represents somewhat of an unfortunate paradox: biomarkers of cell-mediated protection are required for clinical trials, but clinical trials are required to identify cell-mediated biomarkers associated with protection. Clinical trials must move forward with the intent of identifying the biomarkers which best correlate with clinical outcomes in the face of the current uncertainty.

Moreover, key biomarkers of protection will likely come in two types. Some biomarkers of protection may reflect generalizable immunologic responses, indicative of a healthy cell-mediated immune system, but independent of the specific vaccine antigens. These biomarkers could represent red herrings, as they may correlate with positive clinical outcomes, but not truly represent causative correlates of protection. In contrast, other biomarkers will reflect SARS-CoV-2 specific responses that provide vaccine-specific immunological protection. Only through the collection of a broad array of cell-mediated vaccine responses will we be able to distinguish these two types of biomarkers.

Many parameters affect vaccine-induced cellular immunity. These including vaccine modalities, antigen repertoires, adjuvants, delivery routes, doses, and dose intervals. When comparing different vaccines, it is important that we work towards the standardization of assays and methodologies to best deconvolute the relative contributions of these parameters. This will facilitate the optimization of future vaccines and vaccination regimes to achieve optimal cell-mediated immune responses.

Compared to antibody-based approaches, measurements of cellular immunity pre and post vaccination present extra challenges with regards to shipping, handling, and storage logistics, as they require viable cells (38). These logistical challenges are not insurmountable. Measurements of cellular immunity are implemented routinely in clinical trials for a variety of indications (39–44). Profiling of immunological cells at trial screening is absolutely required for CAR-T programs to ensure proper T cell depletion pre-treatment, and proper CAR-T infusion post-treatment (45). Immunological profiling at trial screening is also relatively commonplace for companion diagnostics in the oncology field (39), and evaluation of vaccine efficiency in a highly pre-exposed population is routine in influenza vaccine trials. Recently, results from a phase I clinical trial evaluating a T cell-stimulating peptide vaccine were published (46). This trial demonstrated favourable safety profiles with a broad and potent induction of T cell responses independent of the variant of concern (VoC) status. phase II trials evaluating T cell vaccines in B cell or antibody deficient patients are ongoing.

Still, it must be acknowledged that evaluating cell-mediated immunity on +30,000 participants of phase III clinical trial may not always be feasible. As a starting point, the pre-vaccination immune status of a sub-group of study participants should be evaluated, focusing particularly on immunologically vulnerable demographics such as the elderly. This approach is already in practice for influenza vaccine programs (41, 42). Once this paradigm is established, it should be expanded to include representation from all demographics and immune statuses.

Alternate solutions are also in development, such as wearable sensors capable of correlating vaccine-induced physiological changes to antibody and cell-mediated responses (47), Interferon release assays (48), TCR sequencing systems (49), and skin tests (50). Innovations link these could greatly reduce the cost and complication of evaluating the cell-mediated immune response, rendering these assessments more accessible for clinical trials.

Put simply, the benefits of pre-vaccine immune assessments greatly outweigh the costs. Given the variability and prevalence of previous vaccination and/or SARS-CoV-2 infection, pre-vaccine immune assessments are now essential to the development of second-generation vaccines and the design of their corresponding clinical trials. Moreover, evaluation of cell-mediated immune function must be included, as neglecting to do so will leave questions of vaccine waning, resilience to VoC, and effectiveness in immunocompromised cases unanswered. Without these additions, clinical trial data will be greatly confounded, impairing the ability of regulators and governments to select the most efficacious, robust, and long-lasting vaccines possible.

With the huge incidence of infections accompanying each successive wave of SARS-CoV-2 variants, we have a perfect, yet transient, opportunity to expand our understanding of cell-mediated immunity and apply that understanding to how we protect the most vulnerable members of society through vaccination practice. Unless we study cell-mediated immunity during controlled vaccine trials, we will miss an opportunity that we might not see again until the next pandemic. The opportunity is NOW, and the consequences of missing that opportunity could truly cost millions of lives in the future.

## Data availability statement

The original contributions presented in the study are included in the article/Supplementary Material. Further inquiries can be directed to the corresponding author.

## Author contributions

All authors listed have made a substantial, direct, and intellectual contribution to the work and approved it for publication.

## Funding

The CoVAXCEN consortium is supported by the Robert J. Havey, MD Institute for Global Health’s gift fund at Northwestern University, Feinberg School of Medicine, Arsenal Capital, and the Aimee and Stephen McLean Family Fund.

## Conflict of interest

Authors EP, SSu, TC and DM-S were employed by company CellCarta. Authors TC and JM were employed by company Arsenal Capital. Author SSt was employed by company physIQ.

The remaining authors declare that the research was conducted in the absence of any commercial or financial relationships that could be construed as a potential conflict of interest.

## Publisher’s note

All claims expressed in this article are solely those of the authors and do not necessarily represent those of their affiliated organizations, or those of the publisher, the editors and the reviewers. Any product that may be evaluated in this article, or claim that may be made by its manufacturer, is not guaranteed or endorsed by the publisher.
